# Porcine reproductive and respiratory syndrome virus RNA detection in tongue tips from dead animals

**DOI:** 10.3389/fvets.2022.993442

**Published:** 2022-09-21

**Authors:** Isadora F. Machado, Edison S. Magalhães, Ana Paula S. Poeta Silva, Daniel C. A. Moraes, Guilherme Cezar, Mafalda P. Mil-Homens, Onyekachukwu H. Osemeke, Rodrigo Paiva, Cesar A. A. Moura, Phillip Gauger, Giovani Trevisan, Gustavo S. Silva, Daniel C. L. Linhares

**Affiliations:** ^1^Department of Veterinary Diagnostic and Production Animal Medicine, College of Veterinary Medicine, Iowa State University, Ames, IA, United States; ^2^Iowa Select Farms, Iowa Falls, IA, United States

**Keywords:** PRRSV, surveillance, tongue tips, processing fluids, family oral fluids, pooled serum samples, population-based sampling

## Abstract

The control of porcine reproductive and respiratory syndrome virus (PRRSV) hinges on monitoring and surveillance. The objective of this study was to assess PRRSV RNA detection by RT-PCR in tongue tips from dead suckling piglets compared to serum samples, processing fluids, and family oral fluids. Tongue tips and serum samples were collected from three PRRSV-positive breeding herd farms (farms A, B, and C) of three different age groups: newborns (<24 h), processing (2 to 7 days of age), and weaning (18 to 22 days of age). Additionally, processing fluids and family oral fluids were collected from 2–7 days of age and weaning age, respectively. In farms A and B, PRRSV RNA was detected in tongue tips from all age groups (100 and 95%, respectively). In addition, PRRSV RNA was detected in pooled serum samples (42 and 27%), processing fluids (100 and 50%), and family oral fluids (11 and 22%). Interestingly, the average Ct value from tongue tips was numerically lower than the average Ct value from serum samples in the newborn age. In farm C, PRRSV RNA was only detected in serum samples (60%) and family oral fluids (43%), both from the weaning age. Further, no PRRSV RNA was detected in tongue tips when pooled serum samples from the same age group tested PRRSV RNA-negative. Taken together, these results demonstrate the potential value of tongue tips for PRRSV monitoring and surveillance.

## Introduction

Porcine reproductive and respiratory syndrome virus (PRRSV) is an RNA virus classified into two distinct species, PRRSV-1 (*Betaarterivirus suid* 1) first reported in Europe, and PRRSV-2 (*Betaarterivirus suid* 2), first reported in North America, and today both species share worldwide distribution among commercial swine populations (1–3). Affected breeding herds typically face an increased incidence of abortions, neonatal losses (stillbirths and mummies), pre-weaning mortality, and premature farrowing (4). The total cost of PRRSV infections in the US breed-to-wean and growing-pig herds was estimated at $664 million annually (5), $150 million in Canada (6), and €126 per sow in Dutch sow herds undergoing an 18-week outbreak period (7). Therefore, reducing the number of PRRSV outbreaks in breed-to-wean herds is critical to overcoming losses and improving the sustainability of the swine industry.

Practical and effective monitoring and surveillance strategies to measure PRRSV circulation and shedding are essential to support decisions on disease management practices such as gilt introduction and health interventions. In the last decade, the US swine industry has significantly increased the proportion of population-based sampling for pathogen monitoring compared to individual-based sampling methods (8). The most frequently used population-based sample types are oral fluids (9), processing fluids (10), and family oral fluids (11). It has been reported that population-based methods increase herd sensitivity without increasing cost, time, and labor (10, 12), compared to bleeding a subset of animals.

However, the current population-based sampling schemes commonly used for PRRSV monitoring do not cover all pig production phases, e.g., newborn piglets and gestating sows. In cases in which the application of individual- and population-based samplings are limited, new methods have been proposed to monitor PRRSV infection, such as the usage of “tongue tips” (13). Tongue tip sampling consists of collecting a fragment of tongue tissues from dead animals and could be used in breed-to-wean herds for PRRSV surveillance (13). Given that approximately 36% and 40% of the piglet losses happen during the first and from the second to the seventh day of life, respectively (14), collecting tongue tips from dead pigs for monitoring and surveillance may be an effective way to sample a large number of pigs in the farrowing room. Therefore, the objective of this study was to evaluate the use of tongue tips in commercial breed-to-wean herds for PRRSV detection in three different age groups of suckling pigs (at farrowing, processing, and weaning ages).

## Materials and methods

### Overview and study design

**Figure 1:**
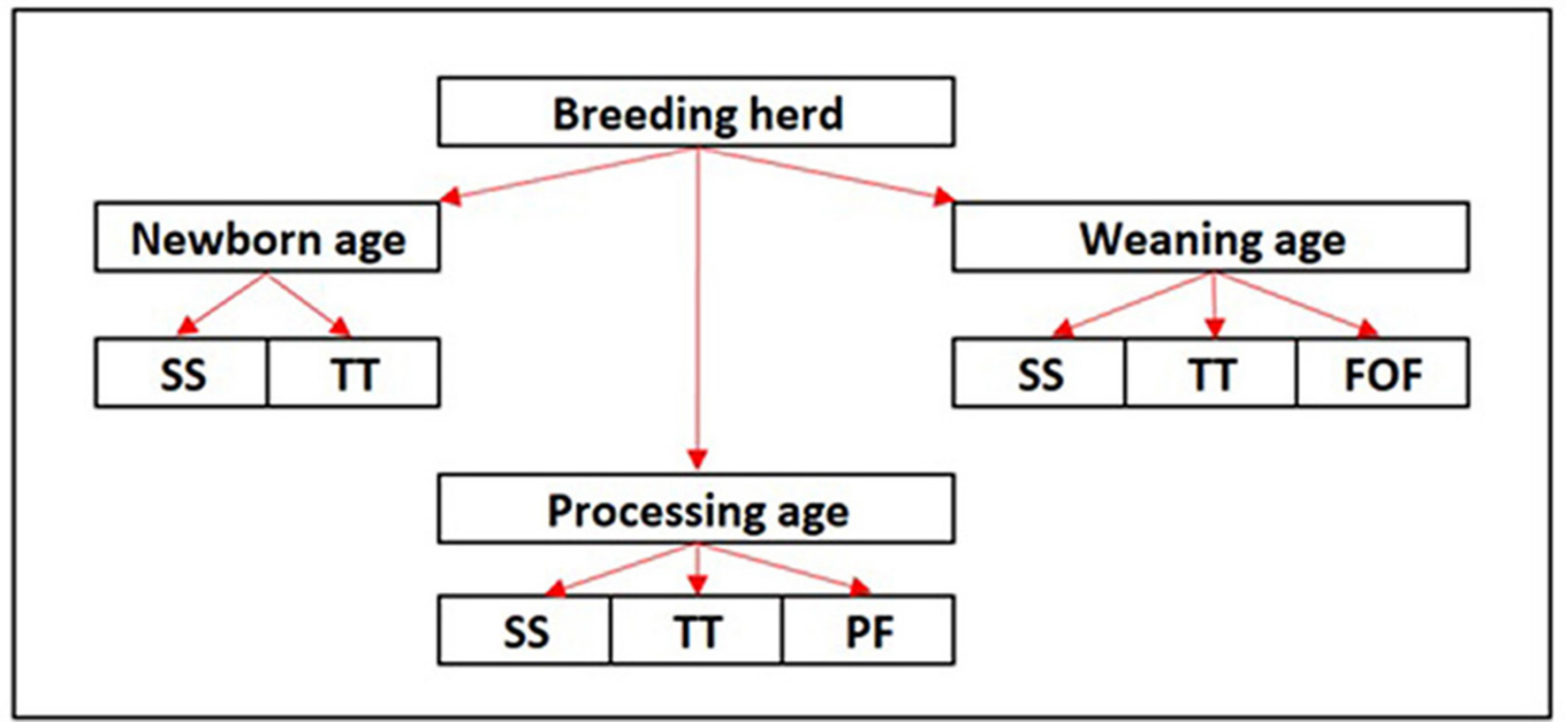
Diagram of the study design. SS, serum sample; TT, tongue tips; PF, processing fluids; FOF, family oral fluids.

This was a cross-sectional field study in which samples were collected from three different breed-to-wean farms (farms A–C) in the midwestern region of the United States (USA) in April of 2022. The farms were conveniently selected based on prior evidence of PRRSV circulation based on diagnostic results and clinical signs. At each farm, the target population for sample collection was classified into three different age groups: newborn age (<24 h of age), processing age (2 to 7 days of age), and weaning age (18 to 22 days of age). Serum samples and tongue tip samples were collected from piglets across all three ages, while processing fluids and family oral fluids were collected from processing age and weaning age animals, respectively (Figure 1).

Tongue tips were collected from dead piglets and were placed in disposable plastic bags separated per age group and per farrowing room, and the number of samples depended on the number of dead animals available in each room during the visit. Within the same room and age group from which tongue tips were collected, one piglet per litter was selected for serum sampling (*n* = 45 samples per age group). Additionally, from the same room in which tongue tips and serum samples were collected, matching processing fluids (*n* = 2 per room) were collected from the processing age group (approximately 2–7 days of age), and family oral fluids (*n* = 15 per room) from the weaning age group (18–21 days of age). Up to 3 days prior to the farm visit, tongue tips from dead animals were also collected by farm personnel from each age group. The procedures described in this study were approved by the Iowa State University Institutional Animal Care and Use Committee ISU-IACUC (IACUC – 19-118).

### Study population

The study breed-to-wean farms were located in Iowa, USA. Farm A (3,500 sows), Farm B (7,000 sows), and Farm C (7,500 sows) were managed in a continuous farrowing system, with an average weaning age of 21 days. All farms were vaccinating the breeding herd for PRRSV using a commercial modified-live PRRSV vaccine and were classified as positive unstable at high prevalence based on Holtkamp et al. (15).

### Sample collection

**Figure 2:**
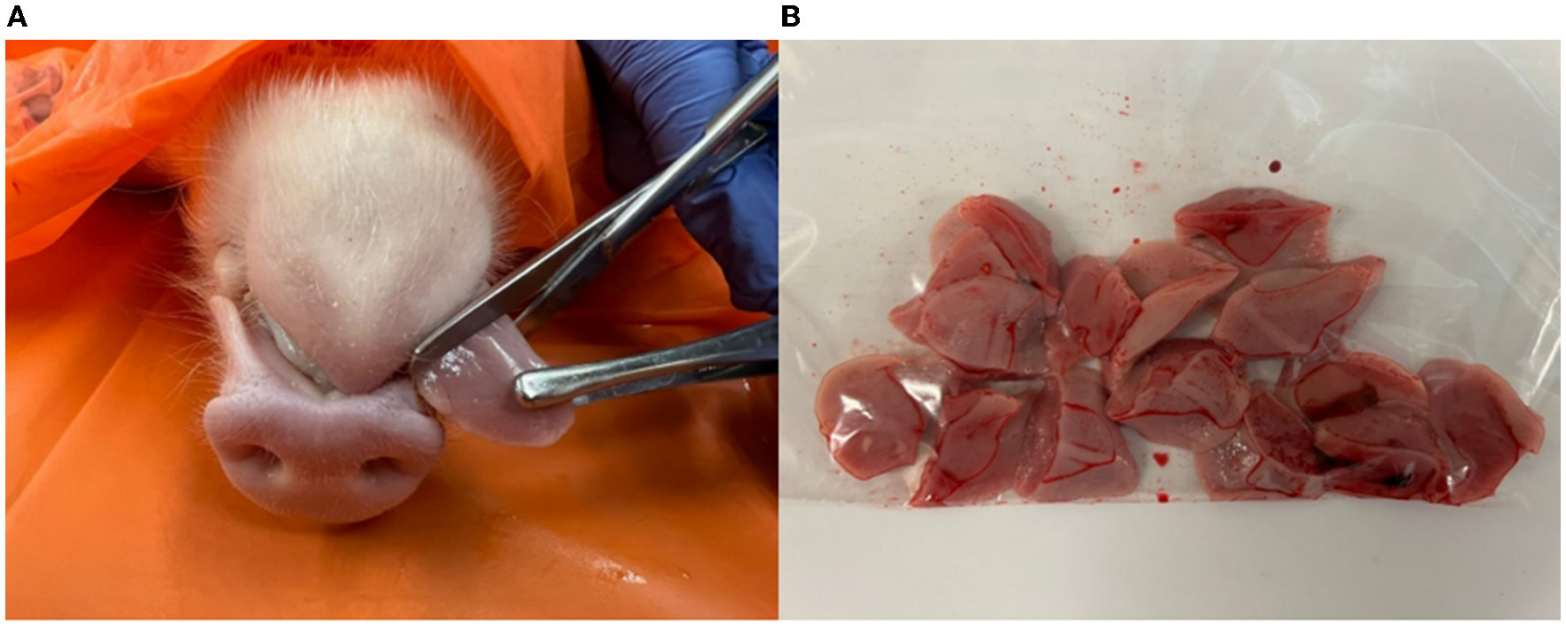
Tongue tip collection. Tongue tips were held from the dead piglets with Russian Tissue Forceps, severed with Mayo Dissecting 6.5” Straight Blunt Scissors (≈ 2 cm) **(A)**, and placed in a disposable plastic bag **(B)**.

The number of samples taken on each farm was based on the available number of litters in each of the three age groups (newborn, processing, and weaning age groups) on the sampling day (Table 1). Tongue tips were held from the dead piglets with Russian Tissue Forceps, severed with Mayo Dissecting 6.5” Straight Blunt Scissors from each age group (approximately 2 cm), and placed in a disposable plastic bag (Figure 2). Following sampling, tongue tips were frozen at −20 °C, as previously described by Baliellas et al. (13).

**Table 1:** Porcine reproductive and respiratory syndrome virus RNA detection by sample type and age group in three commercial breed-to-wean farms.

		**Serum**		**Tongue tips**		**Processing fluids**		**Family oral fluids**	
**Farm**	**Age Group**	**Number of samples and percentage positive^†^**	**Ct average (min-max)**	**Number of samples and percentage positive^‡^**	**Ct average (min-max)**	**Number of samples and percentage positive^‡^**	**Ct average (min-max)**	**Number of samples and percentage positive^‡^**	**Ct average (min-max)**
	Newborn	3/9 (33.3%)	34 (32.6–36.2)	2/2 (100%)	29.3 (24–34.6)	NA	NA	NA	NA
A	Processing	9/17 (53%)	24.4 (16.9–35.8)	5/5 (100%)	27.8 (24.2–31.6)	3/3 (100%)	32.2 (31.3–33.8)	NA	NA
	Weaning	4/12 (33.3%)	33.5 (31.2–36.9)	2/2 (100%)	36.3 (35.7–36.9)	NA	NA	2/17 (11.7%)	34.8 (34.7–35)
	Newborn	3/13 (23%)	26.8 (22.6–29.6)	10/11 (90.9%)	26.4 (21.4–34.7)	NA	NA	NA	NA
B	Processing	2/8 (25%)	19.3 (19.1–19.5)	4/4 (100%)	27.8 (20.3–35.8)	1/2 (50%)	21.9	NA	NA
	Weaning	3/8 (37.5%)	21.6 (19.0–25.0)	6/6 (100%)	29.3 (23.5–33.0)	NA	NA	8/35 (22.8%)	32.4 (25.1–36.8)
C	Newborn	0/11 (0%)	-	0/5 (0%)	-	NA	NA	NA	NA
	Processing	0/8 (0%)	-	0/7 (0%)	-	0/4 (0%)	-	NA	NA
	Weaning	6/10 (60%)	34.8 (32.3–35.5)	0/6 (0%)	-	NA	NA	10/23 (43.4%)	33.1 (30.7–36.3)

† Number positive/total pools tested.

‡ Number positive/total pools tested.

NA, not applicable.

Blood samples from the jugular vein were collected from piglets of all age groups in the same room as tongue tips, using a single-use serum sterile system (Becton, Dickinson and Company, Franklin Lakes, NJ USA).

Processing fluids from the processing age group were collected by the farm personnel, as previously described (10). Briefly, the testicle and tail tissues collected from the processed litters were placed in a disposable plastic bag. At the end of piglet processing, the processing fluids were transferred from the disposable plastic bag to a sterile 50 ml conical plastic tube. The number of processing fluid bags depended on the farm size.

Family oral fluids from weaning age litters were collected using a single strand (0.5 cm diameter), 100% cotton unbleached rope (Web Rigging Supply, Lake Barrington, IL USA), as previously described (11). Briefly, the ropes were suspended (approximately 10 cm from the floor) in the farrowing crate in a position that allowed access to both the sow and her piglets (30 to 60 min of exposure). After the exposure, the ropes were squeezed, and the liquid was transferred to a 50 ml conical plastic tube. Gloves were changed between each sample collection.

Blood, processing fluids, and family oral fluids were refrigerated (4 °C) immediately after collection, while tongue tips were frozen (−20 °C) and submitted for testing within 24 h.

### Sample processing

In the Iowa State University Veterinary Diagnostic Laboratory (ISU VDL), the exudate from tongue tips was extracted by adding 5 mL of PBS in each tongue tip's bag, homogenized, and collected in a 10 ml conical centrifuge plastic tube. The exudate was centrifuged, the supernatant pipetted, and submitted for RT-qPCR testing.

### Diagnostic testing

Tongue tips, processing fluids, and family oral fluids were tested individually for PRRSV RNA using reverse transcription-quantitative polymerase chain reaction (RT-qPCR), while serum samples were tested in pools of five. PRRSV RNA was tested by RT-qPCR (Applied Biosystems TaqMan kit for North American and European PRRSV detection, Thermo Fisher Scientific, Waltham, MA USA). All biological samples were considered positive for PRRSV RNA when the RT-qPCR cycle threshold (Ct) was below 37.

### Data analysis

Data were analyzed descriptively using Microsoft Excel® software. The proportion of positive samples was obtained by dividing the number of positive samples by the total number of collected samples per specimen for each age group and farm.

## Results

The summary of PRRSV RNA detection by farm, age group, and sample type is described in Table 1. Tongue tips were successfully obtained from all age groups, with an average number of 8 tongue tips per bag, ranging from 1 to 40 tongue tips per bag from all collections, e.g., collected by farm personnel and during the farm visit.

In Farm A, the pooled serum samples were RT-qPCR-positive in all the age groups, with an overall positivity of 42.1%. The processing age group had the highest PRRSV RNA-positivity (53%) on serum samples and also had the lowest Ct value, with an average of 24.4. The tongue tips were 100% PRRSV RNA-positive in all age groups and had a Ct value average of 30. As for processing fluids, 100% were PRRSV RNA-positive in the processing age group, with a Ct value average of 32.2, while 11.7% of the family oral fluids in the weaning age group were PRRSV RNA-positive, with an average Ct value of 34.8.

In Farm B, the pooled serum samples were RT-qPCR-positive in all age groups, with an overall positivity of 27.5%. The weaning age group had the highest PRRSV RNA-positivity (37.5%) on serum samples. However, the processing age group had the lowest Ct value, with an average of 19.3. The tongue tips had 95.2% positivity among all age groups and had a Ct value average of 27.5. As for the processing fluids, 50% were PRRSV RNA-positive with an average Ct value of 21.9, while 22.8% of the family oral fluids in the weaning age group were PRRSV-positive, with an average Ct value of 32.4.

In Farm C, the pooled serum samples were RT-qPCR-positive only in the weaning age group (60%), with an average Ct value of 34.8. The tongue tips tested negative for all age groups. As for the processing fluids, all the results were PRRSV RNA-negative, while 43.4% of the family oral fluids in the weaning age group were PRRSV RNA-positive, with a Ct value of 33.1.

## Discussion

This was a cross-sectional study describing tongue tips as an alternative population-based method for PRRSV monitoring and surveillance. It reports similar PCR results between tongue tips, serum samples, processing fluids, and family oral fluids. The objective of this study was not to estimate farm-level prevalence but to demonstrate the use of tongue tips to detect PRRSV RNA in suckling pigs from endemically infected breeding herds. Collecting tongue tissues from dead piglets of different ages in the farrowing room was practical, suitable, and time-efficient for farm personnel under field conditions. Advantages of tongue tip sampling over the individual-based methods include that samples can be collected by a single person, and it does not cause stress to pigs. Furthermore, tongue tips may also be used as an alternative sampling method in farms not performing castration for such reasons as animal welfare claims (16), i.e., when processing fluid sampling is not an option.

The detection of PRRSV RNA in tongue tips was expected due to the characteristics of the tongue's exudate, mainly composed of blood and saliva. In two of the three sampled farms, it was shown that PRRSV RNA was detected in 100% (farm A) and 95.2% (farm B) of collected tongue tip samples, and pooled serum samples were positive in all age groups in different percentages. In contrast, at farm C, no PRRSV RNA detection was observed in tongue tips from all age groups, and pooled serum samples were PRRSV RNA-positive only in the weaning age. PRRSV RNA was generally detected in tongue tips in all age groups in which PRRSV RNA was also detected in serum samples. Further, no PRRSV RNA was detected in tongue tips when serum samples from the same group tested PRRSV RNA-negative. Taken together, these results demonstrate the diagnostic value of tongue tips.

Interestingly, the average Ct value from tongue tips was numerically lower than the average Ct value from serum samples in newborns (<24 h). This finding agreed with Baliellas et al. (13), and it might be explained by the fact that tongue tips were derived from dead animals, which were potentially more likely to harbor PRRSV. Additionally, PRRSV viremia can be detected 6 to 48 h post-infection (17), and PRRSV RNA-positive tongue tips from neonatal pigs were demonstrated in this study to be great indicators of PRRSV activity in the breeding herd and could be an alternative tool to umbilical cords (18), serum, and placenta to characterize vertical transmission in stillbirths.

The potential of tongue tips for PRRSV RNA detection was also evaluated with other population-based methods. It was found that PRRSV RNA detection was similar in tongue tips and processing fluids on farm A. However, PRRSV RNA detection was numerically higher in tongue tips than in processing fluids in processing age pigs on farm B. This might reflect the main characteristic of tongue tips, e.g., it is risk-based sampling while processing fluids is part of the piglet processing routine (healthy or sick animals are sampled). Further, a PCR-positive in processing fluids may indicate both vertical and lateral transmission. Even though RT-qPCR Ct results from tongue tips were numerically lower than processing fluids, the number of tongues was not controlled, and further studies are warranted to characterize PRRSV viral load in tongue tips.

Regarding family oral fluids collected at weaning age, all farms obtained PRRSV RNA-positive results. However, PRRSV RNA was detected in tongue tips in two of three farms (farms A and B). These results suggested that herd sensitivity might differ between these sample types depending on age group. Thus, more studies are needed to compare diagnostic parameters among sample types across different ages.

Baliellas et al. (13) reported that the optimum number of tongue tips per bag was 30 to 100 to obtain enough exudate for PCR testing. However, in this study, tongue tips were collected by room and pig age group, with the number of tongues per bag limited by the number of daily dead pigs. Consequently, the number of tongue tips per bag varied (one to 40 tongues per bag). Thereby, the extraction of the exudate from the tongue tips for the PCR testing was improved with the addition of PBS. Yet, PRRSV RNA was detected in bags containing one tongue tip, as well as in bags containing 40 tongue tips. Further studies estimating the proper number of tongue tips per bag and sample size that maximizes PRRSV detection in pig populations are warranted.

In conclusion, these results showed the potential diagnostic value of tongue tips and supported the use of tongue tips for PRRSV monitoring and surveillance. However, more studies are needed to further elucidate the performance of tongue tips for PRRSV detection in different prevalence scenarios and sample implementation in different veterinary diagnostic laboratories.

## Data availability statement

The original contributions presented in the study are included in the article/supplementary material, further inquiries can be directed to the corresponding author.

## Ethics statement

The animal study was reviewed and approved by Iowa State University Institutional Animal Care and Use Committee ISU-IACUC (IACUC – 19-118). Written informed consent was obtained from the owners for the participation of their animals in this study.

## Author contributions

DL and GS designed the study. IM and EM coordinated the sample and data collection. IM did the formal analyses, designed graphs, and wrote the manuscript. All authors contributed to the sample collection, diagnostic testing, as well as reviewed, and approved the final manuscript.

## Conflict of interest

The authors declare that the research was conducted in the absence of any commercial or financial relationships that could be construed as a potential conflict of interest.

## Publisher's note

All claims expressed in this article are solely those of the authors and do not necessarily represent those of their affiliated organizations, or those of the publisher, the editors and the reviewers. Any product that may be evaluated in this article, or claim that may be made by its manufacturer, is not guaranteed or endorsed by the publisher.
